# Effects of zinc against mercury toxicity in female rats 12 and 48 hours after HgCl2 exposure

**DOI:** 10.17179/excli2015-709

**Published:** 2016-04-12

**Authors:** Mariana Mesquita, Taíse F. Pedroso, Cláudia S. Oliveira, Vitor A. Oliveira, Rafael Francisco do Santos, Cezar Augusto Bizzi, Maria Ester Pereira

**Affiliations:** 1 Post-Graduate Course in Biological Science - Toxicological Biochemistry, Federal University of Santa Maria, Santa Maria, RS, Brazil; 2 Department of Chemistry, Federal University of Santa Maria, Santa Maria, RS, Brazil; 3 Department of Biochemistry and Molecular Biology, Federal University of Santa Maria, Santa Maria, RS, Brazil

**Keywords:** females, nephrotoxicity, acute, mercury, zinc

## Abstract

This work investigated the toxicity of inorganic mercury and zinc preventive effects in female rats sacrificed 12 or 48 h after HgCl_2_ exposure. Female Wistar rats were subcutaneously injected with ZnCl_2_ (27 mg/kg) or saline (0.9 %), and 24 h later they were exposed to HgCl_2_ (5 mg/kg) or saline (0.9 %). Rats sacrificed 12 hours after Hg administration presented an increase in kidney weight and a decrease in renal ascorbic acid levels. Zinc pretreatment prevented the renal weight increase. Rats sacrificed 48 h after Hg exposure presented a decrease in body weight gain, an increase in renal weight, a decrease in renal δ-aminolevulinic acid dehydratase activity, an increase in serum creatinine and urea levels, and a decrease in kidney total thiol levels. Zinc pretreatment partly prevented the decrease in body weight gain and increase in creatinine levels, in addition to totally preventing renal δ-aminolevulinic acid dehydratase inhibition. Mercury accumulation in the kidney and liver in both periods was observed after Hg administration. These results show the different Hg effects along the time of intoxication, and a considerably preventive effect of zinc against Hg toxicity.

## Introduction

Mercury (Hg) is a metal without biological function and an environmental pollutant frequently mentioned for causing toxic effects in living organisms (Mergler et al., 2007[29]; Scheuhammer et al., 2007[46]). Its release can occur through natural sources such as volcanic activities or anthropogenic activities such as industrial processes, agriculture and mining (Li et al., 2009[26]; Magos and Clarkson, 2006[28]), consequently making human exposure to Hg almost impossible to avoid. Mercury is the third most dangerous metal, right after arsenic and lead according to the Agency for Toxic Substance and Disease Registry (ATSDR) (Emsley, 2001[12]). Mercury can be found in two different chemical forms: the organic form, which corresponds to a mercury atom bonded to a carbon atom, and the inorganic form, which includes the elemental form (valence 0) and its oxidized forms (Kim and Zoh, 2012[25]). Elemental mercury and the Hg organic form affect primarily the central nervous system (Farina et al., 2011[15]). Mercury chloride (inorganic form), which is the object of this study, is known as a nephrotoxic agent (Zalups, 2000[52]). It has been reported to cause disorders in the liver (Berlin et al., 2007[4]; Moraes-Silva et al., 2012[30]) as well as in the reproductive and cardiovascular systems (Heath et al., 2009[20]; Zhang et al., 2013[53]), coupled with causing behavioral alterations (Peixoto et al., 2007[37]; Franciscato et al., 2009[18]).

Mercury toxicity is associated to its high affinity for sulfhydryl groups (-SH), forming stable complexes and causing several alterations, such as structural changes of sulfhydryl enzymes and the inactivation of their active sites (Rooney, 2007[42]). Thus, the binding of mercury to -SH groups of antioxidants, for instance glutathione (GSH), reduces the capacity of reactive species (RS) neutralization. The reduction in antioxidant defenses added to the fact that mercury exposure can increase RS levels results in an imbalance in the pro-oxidant/antioxidant system, generating a condition of oxidative stress (Farina et al., 2003[14]; Agarwal et al., 2010[1]). In addition, several studies have shown that inorganic mercury exposure causes changes in body and organ weight, decrease in renal δ-aminolevulinic acid dehydratase (δ-ALA-D) activity, increase in serum urea and creatinine levels, as well as renal histopathological damages (Favero et al., 2014[16]; Franciscato et al., 2011[19]; Oliveira et al., 2014[32]; Peixoto and Pereira, 2007[35]). Furthermore, although the liver is not the preferential target organ, alterations in hepatic enzymes have been observed (Moraes-Silva et al., 2012[30]; Oliveira et al., 2014[32]; Peixoto and Pereira, 2007[35]). 

Among the compounds researched to prevent the damage caused by mercury, natural substances such as vitamin E and omega-3 fat acid have shown beneficial effects (Al-Attar, 2011[2]; Karapehlivan et al., 2014[24]). Reports from our research group have revealed promising effects of ZnCl_2_ in the prevention of biochemical changes induced by mercury chloride in the liver and kidneys of rats (Favero et al., 2014[16]; Franciscato et al., 2009[18]; Moraes-Silva et al., 2012[30]; Oliveira et al., 2014[32]; Peixoto and Pereira, 2007[35]). Zinc is an essential metal, which plays an important role in several biochemical and cellular functions (Fang et al., 2002[13]), and it is a cofactor and structural element of several proteins, participating in the stabilization of biological membranes, replication and transcription of DNA and intercellular signaling. (Sandstead et al., 2007[44]; Shah, 2011[47]; Stehbens, 2003[48]). Furthermore, zinc is an inductor of detoxificant proteins synthesis, such as metallothioneins (Peixoto et al., 2003[36]; Peixoto et al., 2007[38]).

Most of the studies with experimental animals are conducted with male adult rats or pups exposed for short periods of time to mercury (Favero et al., 2014[16]; Moraes-Silva et al., 2012[30]; Oliveira et al., 2014[31]). However, few studies using acute treatments and females have been carried out. Female mercury intoxication is an important field of study, taking into consideration the possible hormonal influence (Ekstrand et al., 2010[10]; Thomas et al., 1982[50]). Recently, Oliveira et al. (2014[32]) showed the effectiveness of zinc pretreatment facing acute mercury chloride intoxication in female rats sacrificed 24 h after the administration of the toxic metal. However, in spite the fact that zinc prevents Hg alterations in the liver and kidneys, some questions still remain. For example, can zinc pretreatment act as a protector few hours after mercury exposure? How long can the toxic effects be visualized? In this context, the purpose of this study was to investigate the toxic effects of mercury in two different intervals after exposure, 12 and 48 hours, as well as the possible zinc protective effect. 

## Materials and Methods

### Chemicals

Reagents were obtained from Sigma Chemical Co. (St. Louis, MO) and standard commercial suppliers. Commercial kits for biochemical dosages were obtained from Kovalent do Brasil Ltda (São Gonçalo/ RJ/ Brazil) or Labtest Diagnóstica S.A. (Lagoa Santa/ MG/ Brazil).

### Animals

Adult female *Wistar* rats (200 ± 20 g) obtained from the Animal House of the Federal University of Santa Maria were transferred to our breeding colony and maintained at a 12 h light/dark cycle and controlled temperature (22 ± 2 °C). Animals had free access to water and commercial food (GUABI, RS, Brazil) and were handled according to the guidelines of the Committee on Care and Use of Experimental Animal Resources, Federal University of Santa Maria, Brazil (096/2011).

### Exposure to metals

**Figure 1:**
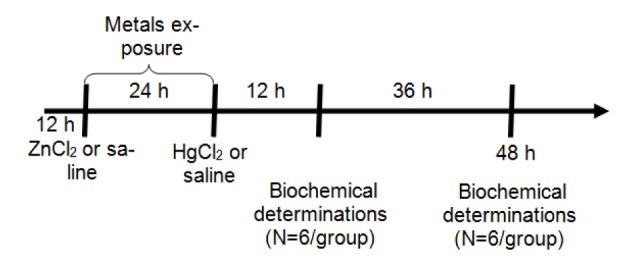
Protocol of exposure: Metals were administered at doses of 27 mg/kg ZnCl_2 _(s.c.) and 5 mg/kg HgCl_2_ (s.c.). Animals were sacrificed 12 h (N = 6) or 48 h (N = 6) after HgCl_2 _exposure for biochemical determinations.

Female *Wistar* rats were distributed on a random basis into four exposure groups (12 animals per group): Sal-Sal, Sal-Hg, Zn-Sal and Zn-Hg. Animals were weighed and subcutaneously (s.c.) injected with 0.9 % NaCl (saline solution) or ZnCl_2_ (27 mg/kg). After 24 h, the animals received saline or HgCl_2_ (5 mg/kg) (s.c.). Half of the animals per group (n = 6) were sacrificed 12 h after HgCl_2_ exposure, and the other half 48 h later (Figure 1(Fig. 1)). Metals were dissolved in saline solution and injected at a volume of 1 mL/kg body weight (b.w.). The doses of ZnCl_2_ and HgCl_2_ were selected according to previous studies performed by Oliveira et al. (2014[32]). It has been reported that ZnCl_2_ in the same dose used in this study caused no damage to the animals (Franciscato et al., 2009[17]).

### Tissue preparation

Twelve or forty-eight hours after the last administration of saline or HgCl_2_, animals were weighed and sacrificed by decapitation. Blood samples were collected in tubes without anticoagulant and centrifuged at 1,050 g for 10 min to obtain the serum, which was used for determination of urea and creatinine levels and alanine aminotransferase (ALT) activity. A portion of the liver was removed and homogenized in a Tris-HCl buffer (10 mM, pH 7.4) containing 1 mM MgSO_4_ for the determination of hepatic ALT activity. For the δ-ALA-D activity assay, blood was collected in tubes with heparin and hemolyzed in distilled water 1:4 (v/v) by agitation in ice bath for 10 min. For the δ-ALA-D activity of tissues and oxidative parameter determination, the kidneys and liver were quickly removed, placed in ice, and homogenized in 5 and 7 volumes of Tris-HCl buffer (10 mM, pH 7.4) with 10 up-and-down strokes at ~1,200 rpm in a Teflon-glass homogenizer, respectively. The homogenate was centrifuged at 3000 *g* for 20 min at 4 °C and the supernatant fraction (S1) was used for analysis. Furthermore, a portion of the kidney, liver and blood was used for determination of mercury and zinc levels.

### Biochemical determinations

#### δ-ALA-D activity 

The enzymatic activity was assayed according to the method of Sassa, (1982[45]), by measuring the rate of product (porphobilinogen - PBG) formation, as previously described (Peixoto et al., 2003[36]). Incubation was started by adding 200 µL of S1 or hemolyzed blood and carried out for 30, 60 and 120 min for the kidney, liver and blood, respectively, at 39 °C. The reaction was stopped by the addition of trichloroacetic acid (TCA) 10 % containing HgCl_2_ 0.05 M; PBG was measured with Ehrlich's reagent, using the molar absorption coefficient of 6.1 x 10^4^ for Ehrlich-PBG salt. The specific enzymatic activity was expressed as nmol of PBG formed per hour per mg protein.

#### Determination of total thiol (TSH) and non-protein thiol (NPSH) levels

Thiol levels from the kidney and liver were determined as previously described by Ellman (1959[11]). For non-protein thiol (NPSH) determination, the protein fraction of 200 μL S1 was precipitated with 200 μL of 4 % TCA (v/v) followed by centrifugation (1,050 *g*, 10 min) and the supernatant used for analysis. The colorimetric test was carried out in a 1 M phosphate buffer, pH 7.4. A curve using glutathione as standard was constructed in order to calculate the SH in the tissue samples. TSH and NPSH levels were expressed as mmol SH per g tissue.

#### Ascorbic acid levels 

Ascorbic acid (AA) determination was performed as described Roe (1954[40]) with some modifications. S1 from the kidney and liver were precipitated in 10 volumes of cold 4 % TCA solution and centrifuged (1,050 *g*, 10 min). An aliquot of the sample in a final volume of 500 µL of the solution was incubated for 3 h at 37 °C; afterwards, 500 µL of H_2_SO_4_ 65 % (v/v) was added into the medium. The reaction product was determined using color reagent containing 4.5 mg/mL dinitrophenyl hydrazine and CuSO_4_ (0.075 mg/mL). Ascorbic acid levels were expressed as µg of AA per g tissue.

#### Alanine aminotransferase activity 

Enzymatic activity was determined by the Thomas (1998[51]) method, using a commercial kit in a medium containing Tris-HCl buffer 55.8 mM pH 7.15, L-alanine 500 mM, 2-Oxoglutarate 15 mM and NADH 0.18 mM, with 50 µL of serum or tissue. The specific enzymatic activity was expressed in serum as U per L and in the liver as U per mg protein.

#### Creatinine

The estimation of serum creatinine levels was performed by measuring the formed product, creatinine picrate. Creatinine was used as standard, utilizing a Labtest commercial kit. The reaction was conducted in a medium containing picric acid 20.2 mM and NaOH 145.4 mM at 37 °C with 50 µL of serum. Levels were expressed as mg of creatinine per dL of serum.

#### Urea

Incubation at 37 °C for 5 min was started by adding 10 µL of serum sample to a medium containing phosphate buffer 19.34 mM pH 6.9, sodium salicylate 58.84 mM, sodium nitroprusside 3.17 mM, and urease (≥ 12.63 UK/L), using a Labtest commercial kit. The reaction was stopped by adding oxidant solution (final concentrations: NaOH 0.07 M and sodium hypochlorite 3.01 mM), and the mixture was incubated for 5 min to achieve color development. Levels were expressed as mg of urea per dL of serum.

### Protein determination

Protein concentrations were determined by the Coomassie blue method using bovine serum albumin as standard (Bradford, 1976[6]).

### Determination of metal levels 

Mercury and zinc levels were determined by ICP OES using an axial view configuration spectrometer (Spectro Ciros CCD, Spectro Analytical Instruments, Kleve, Germany). The samples of wet tissue (about 0.25 g of the kidney and liver and 0.5 mL of blood) were placed in vials and frozen at -18 °C until analysis. Samples were digested with concentrated HNO_3_ in a water bath (100 °C) for 6 h. After digestion, samples were diluted with deionized water to 20 mL and metals were determined by ICP OES. The spectral lines monitored were 194.227 nm and 184.950 nm for Hg and Zn, respectively. 

### Statistical analysis

Results were analyzed by one-way analysis of variance (ANOVA) followed by Duncan's multiple range test when necessary. Results were considered significant when p ≤ 0.05. 

## Results

### Body and tissue weight

Body weight gain and organ weights are shown in Table 1(Tab. 1). One-way ANOVA revealed significant alterations in kidney weight [F(3,20) = 4.854; p ≤ 0.011], but not in liver weight and body weight gain of rats sacrificed 12 h after the intoxication. Hg-exposed rats presented increase in kidney weight, which was prevented by zinc pre-treatment. For rats sacrificed 48 h after mercury exposure, one-way ANOVA revealed alterations in body weight gain [F(3,20) = 3,213; p ≤ 0.045] and in kidney weight [F(3,20) = 9.054; p ≤ 0.001]. Mercury administration decreased the body weight gain and increased the kidney weight. Zinc pre-treatment partially prevented alterations in body weight gain, but not in kidney weight.

### δ-ALA-D activity

**Figure 2:**
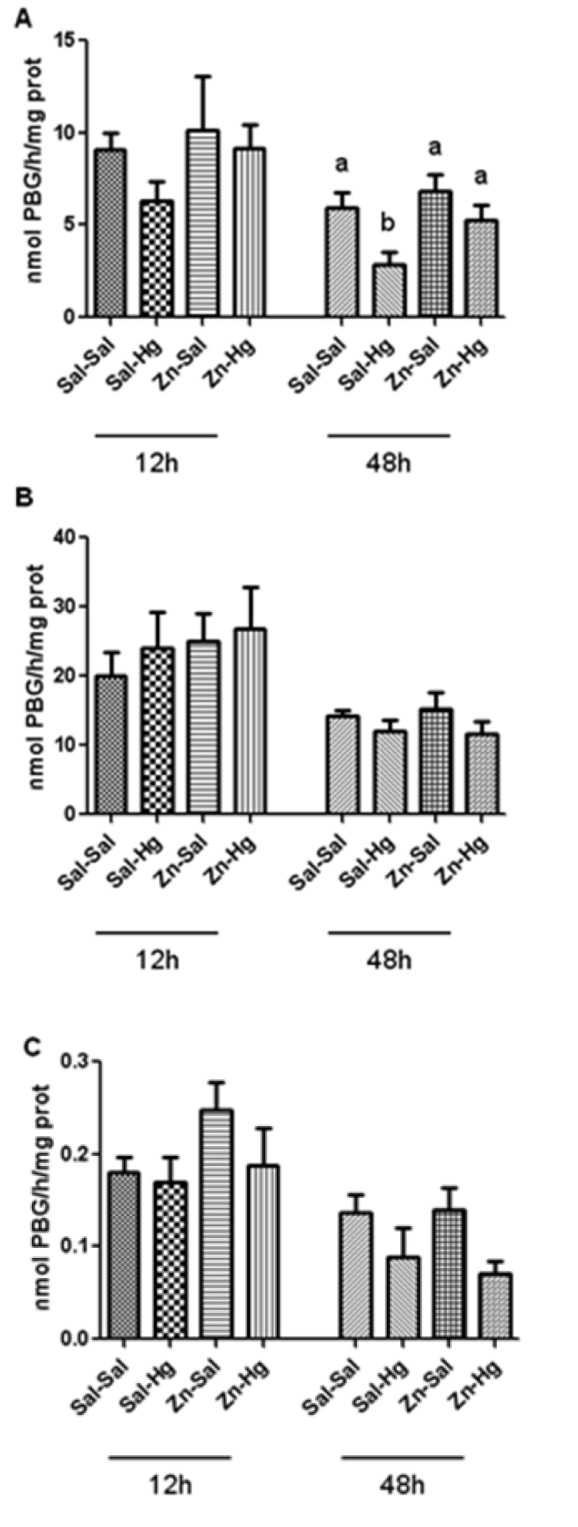
δ-ALA-D activity from the kidney (A), liver (B) and blood (C) of rats treated as described in the Table 1 legend. Results were analyzed by one-way analysis of variance (ANOVA) followed by Duncan's post-hoc test: groups followed by different letters are statistically different (p ≤ 0.05) and groups followed by the same letters are statistically equal (p ≤ 0.05). Data are presented as mean ± S.E.M. (N = 6).

Figure 2(Fig. 2) shows the δ-ALA-D activity from the kidney (A), liver (B) and blood (C). Only renal enzyme activity of rats sacrificed 48 h after intoxication was significantly altered by treatments [F(3,20) = 4.624; p ≤ 0.013]; δ-ALA-D inhibition induced by Hg was prevented by zinc pre-treatment.

### Creatinine and urea levels

Serum creatinine and urea levels are shown in Table 2(Tab. 2). No alterations were observed in urea and creatinine levels when rats were sacrificed 12 h after mercury administration. However, after 48 h of Hg intoxication, a significant increase in creatinine [F(3,20) = 15.659; p ≤ 0.001] and urea [F(3,20) = 15.069; p ≤ 0.001] levels was observed. Zinc pre-treatment partially prevented the Hg effect on creatinine levels. 

### Alanine aminotransferase (ALT) activity

Serum and hepatic ALT activities are shown in Table 2(Tab. 2). Only hepatic ALT activity was altered by treatment in rats sacrificed 48 h after intoxication [F(3,20) = 6.375; p ≤ 0.003]. Although mercury *per se* did not cause any effects, hepatic ALT activity was significantly increased in both groups exposed to zinc.

### Ascorbic acid levels 

**Figure 3:**
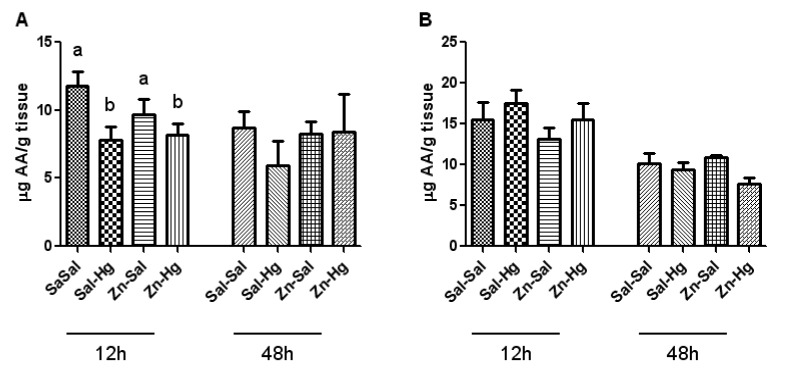
Ascorbic acid levels in the kidney (A) and liver (B) of female rats treated as described in the Table 1 legend. Results were analyzed by one-way analysis of variance (ANOVA) followed by Duncan's post-hoc test: groups followed by different letters are statistically different (p ≤ 0.05) and groups followed by the same letters are statistically equal (p ≤ 0.05). Data are presented as mean ± S.E.M. (N = 6).

Figure 3(Fig. 3) shows the kidney (A) and liver (B) ascorbic acid levels. Only the renal ascorbic acid levels from rats sacrificed 12 h after intoxication presented significant treatment effect [F(3,20) = 3.230; p ≤ 0.044]. Mercury exposure caused a decrease in renal ascorbic acid levels; however, zinc pretreatment did not protect this alteration caused by Hg.

### Total thiol (TSH) and non-protein thiol (NPSH) levels

**Figure 4:**
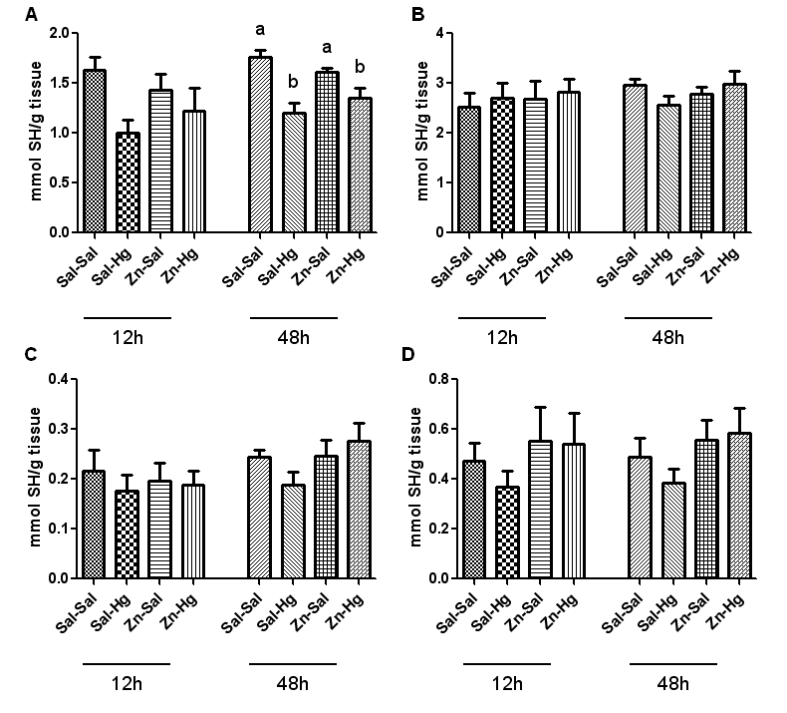
Total and non-protein SH levels in the kidney (A-C) and liver (B-D) of female rats treated as described in the Table 1 legend. Results were analyzed by one-way analysis of variance (ANOVA) followed by Duncan's post-hoc test: groups followed by different letters are statistically different (p ≤ 0.05) and groups followed by the same letters are statistically equal (p ≤ 0.05). Data are presented as mean ± S.E.M. (N = 6).

Total and non-protein SH levels from the kidney (A, C) and liver (B, D) are shown in Figure 4(Fig. 4). One-way ANOVA exhibited a significant treatment effect in total thiol levels in the kidney (A) [F(3,20) = 8.950; p ≤ 0.001] of rats sacrificed 48 h after the end of HgCl_2_ exposure. The treatment with mercury caused a decrease in kidney total thiol levels, and the zinc pre-treatment did not prevent this alteration. No alteration was verified 12 h after mercury exposure.

### Kidney, liver and blood mercury and zinc levels 

Mercury and zinc levels are presented in Table 3(Tab. 3). One-way ANOVA revealed a significant treatment effect in Hg levels in the kidney [12 h F(3,8) = 6.812; p ≤ 0.014; 48 h F(3,8) = 12.960; p ≤ 0.002] and liver [12 h F(3,8) = 4.672; p ≤ 0.036; 48 h F(3,8) = 27.394; p ≤ 0.001]. Animals exposed to mercury presented an increase in kidney and liver Hg levels when compared to the control group in both periods after exposure. Zinc pre-exposure did not alter Hg or zinc levels.

## Discussion

The purpose of this research was to investigate the appearance of the Hg toxic effect in two intervals (12 and 48 h) after mercury exposition. Moreover, we evaluated the preventive effect of zinc chloride against mercury intoxication. 

Mercury treatment caused alterations in renal weight of female rats both times after Hg exposure. The precise mechanism involved in this effect is yet unclear. However, it is known that at a short period Hg can cause several modifications in the kidney, such as cellular pathologies in proximal straight tubules (Homma-Takeda et al., 1999[21]; Zalups, 2000[52]), which could be related to the increase of renal weight (Peixoto et al., 2003[36]). Zinc pre-treatment prevented renal weight increase in rats sacrificed 12 h after Hg exposure; however, at 48 h, the zinc preventive effect was not statistically significant. 

Mercury has high affinity to sulfhydryl groups, which is an important factor of its toxicity (Rooney, 2007[42]). Among the Hg targets, δ-ALA-D is an enzyme that has wide distribution in organisms (Jaffe, 1995[22]). δ-ALA-D has sulfhydryl residues susceptible to binding with Hg (Peixoto et al., 2003[36]; Roza et al., 2005[43]), thereby, δ-ALA-D is an important bioindicator of heavy metal exposure (Oskarsson and Fowler, 1985[33]). In fact, the renal enzyme was inhibited 48 h after exposure, probably because kidneys are the primary target of inorganic mercury accumulation (Zalups, 2000[52]). Twelve hours after intoxication, we verified a light decrease in this enzyme activity induced by Hg. Although renal uptake and accumulation of Hg occurs quickly (Zalups, 2000[52]) and 12 h after Hg exposure the renal levels are high, the action of Hg on kidneys was not sufficient to cause a significant inhibition in δ-ALA-D activity. However, this alteration caused by Hg becomes visible in 24 h (Oliveira et al., 2014[32]) along with this inhibition persisting for 48 h after exposure. Notably, zinc pre-exposure prevented δ-ALA-D activity inhibition. Previously, we had demonstrated the protection by zinc in young and adult rats exposed to mercury (Favero et al., 2014[16]; Franciscato et al., 2011[19]; Oliveira et al., 2014[32]; Peixoto and Pereira, 2007[35]). The preventive effect of Zinc seems to be associated with its ability to induce metallothionein syntheses (Peixoto et al., 2003[36], 2007[38]), which are detoxification proteins able to sequester toxic metals (Romero-Isart and Vasak, 2002[41]).

Creatinine and urea are the main indicators of acute renal damage (Edelstein, 2008[9]), revealing increased levels in the blood in circumstances of renal function damage (Ravel, 1997[39]). Serum creatinine levels are commonly accepted to measure renal function in clinical practice, concerning the glomerular filtration rate (Stevens and Levey, 2005[49]). In regards to these renal markers of toxicity, our study showed an increase in creatinine (around 7.6 times) and urea (4 times) levels in serum of rats sacrificed 48 h after Hg exposure. This result agrees with previous studies of our research group (Franciscato et al., 2011[19]; Oliveira et al., 2014[32]; Peixoto and Pereira, 2007[35]) reinforcing inorganic mercury as a nephrotoxic agent. Zinc pre-exposure prevented partially the increase in creatinine levels, appointing better renal condition when compared to the mercury group.

The determination of ALT activity is a biochemical parameter commonly used as a marker of hepatic damage (Liu et al., 2008[27]). In this study, we verified that the activity of this enzyme in the serum and liver was not altered in either period analyzed after exposure. This result suggests the absence of hepatotoxicity and agrees with other studies that presented similar results using another model of Hg exposure (Oliveira et al., 2014[32]; Peixoto and Pereira, 2007[35]). However, other parameters are required in order to discard this possibility. The pre-treatment with zinc caused an increase in hepatic ALT activity in rats sacrificed 48 h after Hg intoxication. Nonetheless, this alteration does not define a condition of hepatotoxicity, given that serum levels remain unchanged.

Considering the high affinity of Hg by sulfhydryl groups and the effects that it causes in several components of the antioxidant system (Clarkson, 1997[7]; Farina et al., 2003[14]), in addition to excessive formation of free radicals (Pal and Gosh, 2012[34]), some studies have associated the toxic effects of this metal to stress oxidative (Boujbihaa et al., 2009[5]; Agarwal et al., 2010[1]). Regarding the endogenous non-enzymatic antioxidant system, it is important to highlight the ascorbic acid, which is a water-soluble antioxidant of fast action (Jiraungkooskul and Sahaphong, 2007[23]), as well as non-protein thiols (Apel and Hirt, 2004[3]). In the present study, we verified a decrease in renal ascorbic acid levels caused by Hg exposure in rats sacrificed 12 h after intoxication. When these levels are analyzed in a longer period after Hg exposure, such as 24 h (Oliveira et al., 2014[32]) or 48 h, no alteration is found. On the other hand, kidney total thiol levels are decreased 48 h after intoxication in animals that received mercury. Thus, the decrease of ascorbic acid levels may be related with the consumption of this antioxidant in the first moment of intoxication, headlining a change in the antioxidant profile consumed afterwards.

As for mercury deposition, rats exposed to Hg presented an accumulation in the kidneys and liver, and zinc pre-exposure did not alter this accumulation. It is known that zinc induces metallothionein synthesis (Peixoto et al., 2003[36], 2007[38]), and this protein binds to mercury, reducing its availability and effects (Dabrio et al., 2002[8]). Thus, we suggest that despite the high Hg levels found in the kidney, the metal may be partially in inactive forms, due to binding to metallothioneins. Furthermore, the level of Hg found in the liver was not enough to cause hepatic damage considering the parameters analyzed. 

## Conclusion

In conclusion, these results show that mercury causes different alterations in the two periods studied. Rats sacrificed 12 h after mercury exposure did not present significant alterations in most of the parameters analyzed. However, in a longer period after exposure, 48 h, mercury caused changes in almost all parameters analyzed. The zinc pre-treatment showed its effects to prevent some of parameters altered by mercury exposure. 

## Acknowledgements

The authors would like to thank the Conselho Nacional de Desenvolvimento Científico e Tecnológico (CNPq), Coordenação de Aperfeiçoamento de Pessoal de Nível Superior (CAPES).

## Conflict of interest

The authors declare no conflict of interest.

## Figures and Tables

**Table 1:**
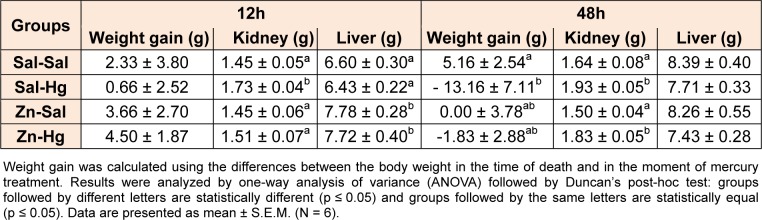
Body weight gain and liver and kidney weights of female adult rats treated (s.c.) with one dose of saline or ZnCl_2_ (27 mg/kg) followed by one dose of saline or HgCl_2_ (5 mg/kg) 24 h later, and killed 12 or 48 h after HgCl_2_ administration

**Table 2:**
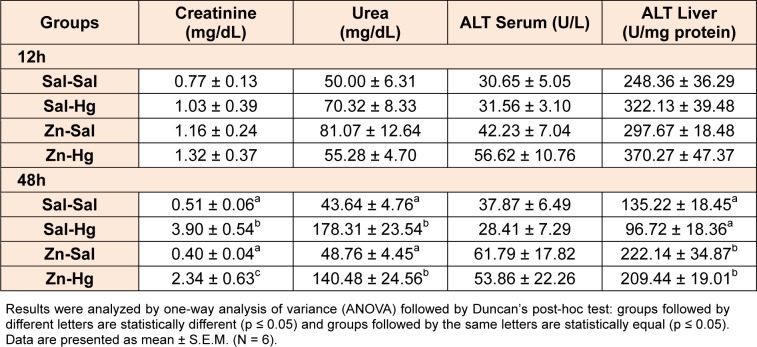
Serum creatinine and urea levels, and serum and hepatic ALT activity of female rats treated as described in the Table 1 legend.

**Table 3:**
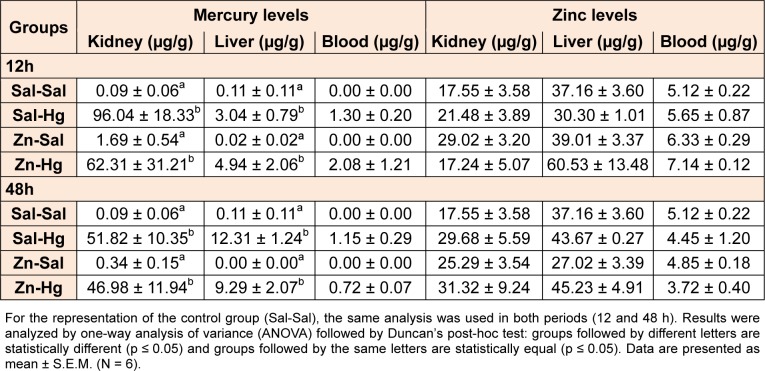
Levels of mercury and zinc in the kidney, liver and blood of female rats treated as described in the Table 1 legend
